# Diagnosis and management of malignant peripheral nerve sheath tumors: Current practice and future perspectives

**DOI:** 10.1093/noajnl/vdz047

**Published:** 2019-11-14

**Authors:** Bethany C Prudner, Tyler Ball, Richa Rathore, Angela C Hirbe

**Affiliations:** 1 Division of Medical Oncology, Department of Medicine, Washington University, St. Louis; 2 Neurofibromatosis Center, Washington University, St. Louis MO; 3 Siteman Cancer Center, Washington University, St. Louis

**Keywords:** diagnosis, MPNST, neurofibromatosis, treatment

## Abstract

One of the most common malignancies affecting adults with the neurofibromatosis type 1 (NF1) cancer predisposition syndrome is the malignant peripheral nerve sheath tumor (MPNST), a highly aggressive sarcoma that typically develops from benign plexiform neurofibromas. Approximately 8–13% of individuals with NF1 will develop MPNST during young adulthood. There are few therapeutic options, and the vast majority of people with these cancers will die within 5 years of diagnosis. Despite efforts to understand the pathogenesis of these aggressive tumors, the overall prognosis remains dismal. This manuscript will review the current understanding of the cellular and molecular progression of MPNST, diagnostic workup of patients with these tumors, current treatment paradigms, and investigational treatment options. Additionally, we highlight novel areas of preclinical research, which may lead to future clinical trials. In summary, MPNST remains a diagnostic and therapeutic challenge, and future work is needed to develop novel and rational combinational therapy for these tumors.

**Key Points**

1. MPNSTs are aggressive soft tissue sarcomas that remain a challenge to diagnose and treat.
2. Multimodality treatment at an experienced center is recommended, with the ability to undergo surgery with negative margins being the only potentially curative therapy.
3. Treatment of metastatic disease is limited to cytotoxic chemotherapy or clinical trials; the hope is that current research will lead to the design of more rational clinical trials.

**Importance of the Study**

This manuscript provides an up to date review of the molecular etiology underlying MPNST development and progression. Additionally, we review the pathologic and imaging considerations associated with diagnosis of the aggressive tumor. Finally, we discuss the current management of localized and metastatic disease with a comprehensive review of clinical trials to date as well as a discussion of the current preclinical research.

Neurofibromatosis (NF1) is one of the most common cancer predisposition syndromes, affecting approximately 1 in 2500 individuals worldwide. The deadliest cancer arising in individuals with NF1 is the malignant peripheral nerve sheath tumor (MPNST).^1^ These malignancies represent approximately 5% of the 15,000 soft tissue sarcomas diagnosed in the United States each year. The main risk factors for the development of MPNST are existing benign plexiform neurofibromas (PNs),^2^ prior radiation treatment,^3^ and large germline mutations involving the entire *NF1* gene (microdeletions) and surrounding genes.^4^ In this review, we will discuss the pathophysiology, diagnostic workup, current treatment options, and clinical trials for MPNST. Additionally, we will discuss new areas of research that may lead to improvements in the diagnosis and treatment of these aggressive cancers.

## Pathophysiology

**Figure 1.**
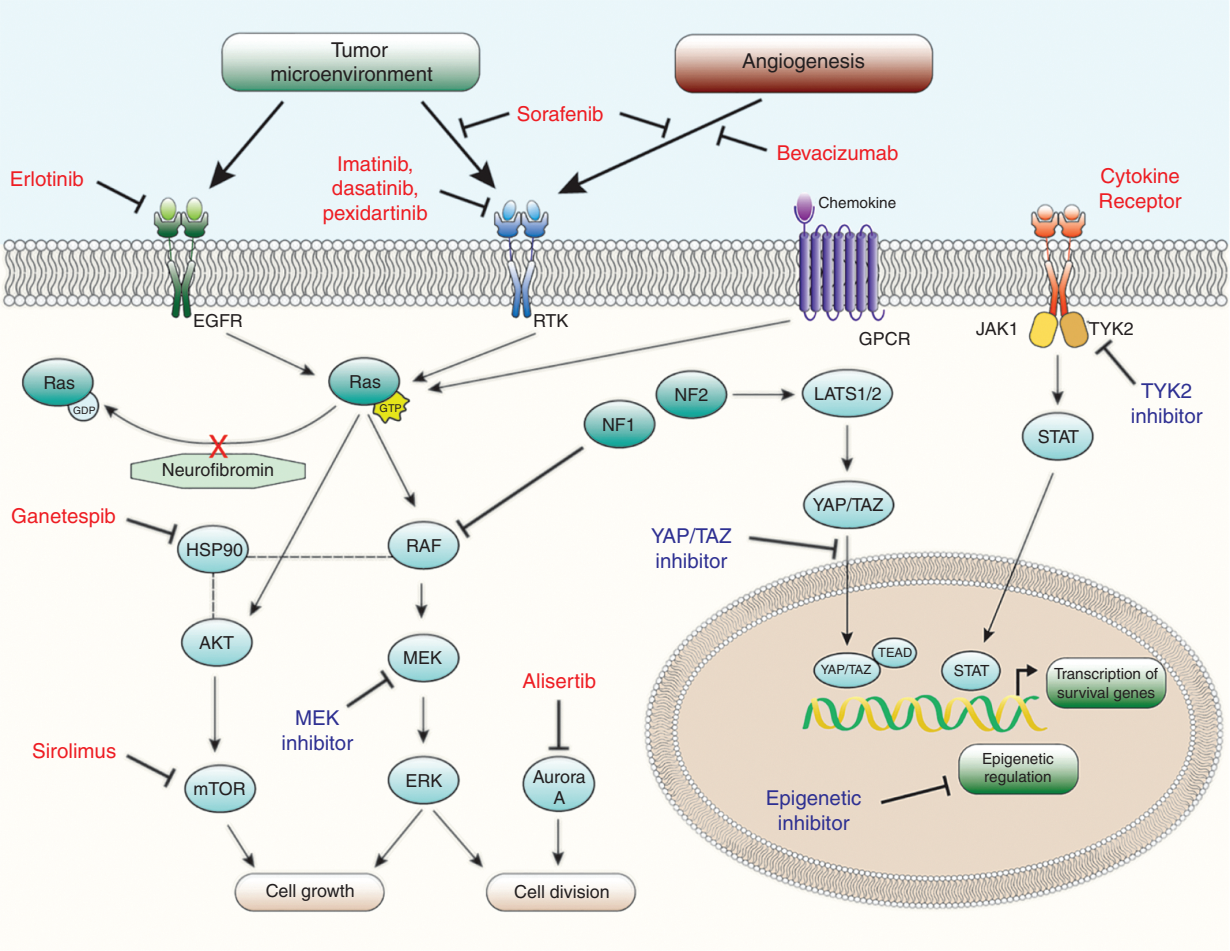
Neurofibromin is a negative RAS regulator. Growth factor binding to cognate receptor tyrosine kinases (EGFR, RTK) or chemokine binding to G-protein coupled receptors (GPCR) lead to activation of RAS and subsequent phosphorylation of downstream RAS effectors, including AKT (mTOR) and RAF (MEK/ERK). Neurofibromin functions in part as a RAS-GTPase activating-related protein that stimulates inherent GTPase activity of RAS, increasing the conversion of active GTP-RAS to inactive GDP-RAS. Loss of neurofibromin leads to increased RAS/RAF effector activity, and greater cell growth. Signals from the microenvironment, HIPPO pathway, Janus kinases, epigenetic regulators, and protein stability pathways also contribute to malignant cell growth. Drugs that have been tested in clinical trials for MPNST are depicted in red alongside their respective targets. Potential drug targets to include in novel combinations for MPNST are depicted in blue alongside the respective targets.

NF1 can be caused by inherited or de novo mutations in the *NF1* gene, which encodes for neurofibromin, a 220 kDa cytoplasmic protein with regions containing homology to GTPase-activating proteins (GAPs). Neurofibromin has subsequently been identified as a GAP for the RAS family of proto-oncogenes. Thus, disruption of *NF1* leads to hyperactive RAS signaling and promotes cell growth.^5^ As a result of the loss of GTPase activity in NF1, the GTP-bound form of RAS dominates, recruiting the serine/threonine protein kinase RAF to activate MEK and ERK.^6^ Additionally, activated RAS leads to downstream activation of PI3K/AKT/mTOR. Together, these pathways lead to stimulation of downstream activators of cell growth, survival, and proliferation (Figure 1).

MPNST is comprised of neoplastic Schwann cells and, in the setting of NF1, most often arise from a benign precursor lesion, termed PN. PN develop in approximately 30–50% of patients with NF1, where they can extend into surrounding structures and cause significant pain. These lesions tend to grow most rapidly during the first decade of life and, when identified early, are monitored for signs of malignant transformation.

**Figure 2.**
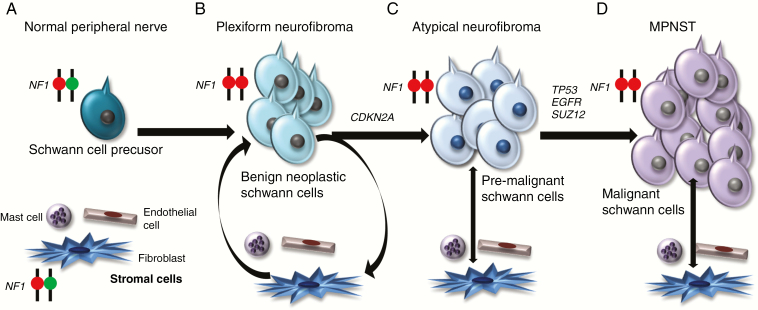
Genomic Evolution of NF1-MPNST. (A) Patients with NF1 start life with one mutant and one normal copy of the *NF1* gene in the cells within their body. (B) Preneoplastic Schwann cell precursors undergo somatic *NF1* loss, resulting in bi-allelic *NF1* inactivation and benign neurofibroma formation. Factors in the *NF1* heterozygous microenvironment also influence tumor formation through the secretion of growth factors, chemokines, and inflammatory mediators. (C) Loss of *CDKN2A* leads to atypical neurofibroma (AN) formation, and (D) mutations in other genes, including *TP53*, *EGFR*, and *SUZ12,* lead to MPNST formation.

While *NF1* gene inactivation and loss of neurofibromin expression characterize the majority of MPNST,^7^ bi-allelic *NF1* loss is insufficient for malignant transformation. This is supported by genetically engineered mouse studies, in which conditional *Nf1* gene inactivation in Schwann cell precursors results in PN development,^8–10^ whereas MPNST formation requires additional genetic alterations. In both mouse and human MPNST, mutations or copy number alterations in genes such as *TP53*, *CDKN2A*, *EGFR*, and *SUZ12* have all been reported as secondary cooperating mutations facilitating malignant progression.^11–15^ In this regard, alterations in *TP53*, *EGFR*, and *SUZ12* are common in MPNST. However, mutations in these genes do not occur in benign PN or atypical neurofibromas (AN),^16–19^ suggesting that these alterations represent later steps in progression. In contrast, *CDKN2A* loss has been reported in as many as 94% of AN.^16,19^ Taken together, these findings support a model in which *CDKN2A* loss occurs during the transition from benign PN to AN, whereas *TP53*, *EGFR*, and *SUZ12* alterations promote evolution to MPNST (Figure 2).

Preclinical mouse studies also show the importance of the *Nf1* heterozygous tumor microenvironment in the formation and maintenance of PN,^8^ where growth factors, chemokines, and inflammatory mediators may accelerate transformation from PN to MPNST.^20^ For example, *Nf1* heterozygous Schwann cells produce c-KIT ligand stem cell factor (SCF) which attracts mast cells,^21^ as well as transforming growth factor beta (TGF-β) which attracts fibroblasts.^22^ These recruited cells in turn secrete other factors, such as platelet derived growth factor (PDGF)^23^ and vascular endothelial growth factor (VEGF),^24^ enhancing tumor cell growth. Continued recruitment and secretion establishes an oncogenic cycle, allowing the neoplasm to expand. Additionally, hematopoietic cells, including macrophages, have begun to emerge as an important signal for MPNST transformation and survival.^25,26^ Furthermore, autocrine loop signaling pathways such as CXCR4/CXCL12 have been implicated in progression of MPNST.^27^ Given these findings, a complex network between a diverse number of cells and signaling pathways is necessary for the development, maintenance, and progression of MPNST.

## Diagnosis

### Imaging

Establishing an accurate diagnosis represents a major challenge in managing patients with NF1-associated PN, AN, and MPNST. A wealth of data has come from the National Cancer Institute (NCI) NF1 Natural History Study as patients are followed over time with serial exams, whole body MRI (WB-MRI) with volumetric analysis, and FDG-PET. The use of WB-MRI offers several potential benefits. First, total tumor burden in NF1 patients can be assessed, as WB-MRI allows for the identification of all PN, including internal PN, which cannot be monitored by physical examination. Given that increased tumor burden is correlated with the risk of developing an MPNST, this information is clinically useful.^28^ Second, the use of WB-MRI has allowed us to follow the natural course of PN development over time, which has expanded our understanding of the progression of PN to MPNST. Studies from both the NCI and the University of Hamburg suggest that most PN growth occurs in children, and significant growth acceleration is uncommon in adults. Furthermore, these tumors typically develop during childhood and rarely develop in adolescence or adulthood.^29,30^

One study utilizing data from the NCI NF1 Natural History Study evaluated 154 patients with NF1 followed over time with imaging. Data from this study suggests that up to 50% of patients with PN develop well-demarcated nodular areas within their PN, termed distinct nodular lesions (DNLs). Most DNLs are larger than 3 cm in size, lack the central dot sign characteristic of PN, and typically show more rapid growth than PN. As such, DNLs often correlate with areas of pain. Tumors with concerning findings (distinct nodular areas/well demarcated, >3 cm, lacking the central dot sign) associated with rapid growth or symptoms underwent biopsy or resection. Of the 154 patients, 24 patients underwent biopsy/resection and there were 17/24 (70%) confirmed AN. Of the confirmed AN, all appeared as DNLs on MRI and were associated with modest FDG uptake (median standard uptake value [SUV] = 7.2).^31^

The Response Evaluation in Neurofibromatosis and Schwannomatosis (REiNS) International Collaboration WB-MRI Working Group has recently published a summary of all available studies involving the use of WB-MRI in this patient population in order to identify the knowledge gaps and guide future studies involving this imaging modality.^32^ Future work is needed to determine the optimal magnet strength, image acquisition protocols, imaging frequency, value of functional imaging, and contrast administration, as well as to test interobserver performance to standardize this imaging modality across institutions. Additionally, further studies are needed to assess whether screening with WB-MRI can reduce the incidence of MPNST in this high risk population.

Despite these advances, MRI is still unable to reliably differentiate between benign and malignant tumors.^33^ Numerous reports have suggested that FDG-PET is more sensitive in the detection of MPNST, and has more significant utility for making a diagnosis than MRI,^33,34^ with sensitivities nearing 90%. With FDG, tumors with SUVs less than 2.5 were correlated with benign lesions on pathology, whereas those with SUVs greater than 3.5 were most often MPNST (26/29; 90%).^34^ The clinical significance of tumors with SUVs between 2.5 and 3.5 remains unclear,^34^ but these patients warrant continued close surveillance. Of note, these criteria are based on delayed imaging at 4 h, which is not a standard practice. While the prior studies mentioned involve imaging of symptomatic patients, there is also emerging data that FDG-PET could be useful in detecting tumors in asymptomatic patients, leading to early detection and improved outcomes. In this study, an SUV cutoff of 3.5 was used to identify lesions at risk. This cutoff was 100% sensitive, but only 49.5% specific in diagnosing malignancy, as the SUV max values of asymptomatic lesions, judged as suspect and retrospectively graded as benign (range 1.81–8.54), largely overlapped with the SUV max of truly malignant lesions (3.16–6.83).^35^ Future studies are beginning to evaluate the utility of combined PET-MRI to improve the sensitivity of these diagnostic strategies.^36^

### Pathology

In contrast to some soft tissue sarcomas, there is no pathognomonic genetic alteration or immunohistochemical stain to diagnose MPNST. Given the lack of specific morphological criteria or immunohistochemical/molecular tests, it can be difficult to distinguish MPNST from other sarcomas. When the gross specimen clearly arises from a nerve, the diagnosis is clear. However, if this is not the case, a variety of immunohistochemical stains may be required to distinguish between sarcomas of muscle, vascular, or other non-neural tissue. Negative staining for cytokeratins and melanoma markers such as Melan-A, MITF, or HMB45 can be helpful in distinguishing MPNST from carcinomas and melanoma, respectively. S100, a Schwann cell marker, is decreased, or completely lost in the undifferentiated state of MPNST. Additionally, in a subset of MPNST, positive p53 immunoreactivity may be identified, indicative of mutant p53 and malignant transformation. Genomic analyses demonstrating *NF1* or *CDKN2A* loss, which are thought to occur early in disease progression, are also suggestive of a diagnosis of MPNST over other malignancies. While this testing may be useful to identify and characterize these tumors,^37–39^ there is no standardized set of immunohistochemical markers that have been applied across clinical laboratories to diagnose MPNST.

MPNST can also be difficult to distinguish from benign counterparts or premalignant precursor lesions, given the continuum from PN to AN to fully developed MPNST. Recent discussions among experts have attempted to define benign and malignant entities, leading to the introduction of a new precursor entity, termed atypical neurofibromatous neoplasm of uncertain biologic potential (ANNUBP)^40^ (Table 1). Additionally, unlike other sarcomas, the distinction between low-grade and high-grade MPNST has uncertain prognostic value when estimating survival, as the only data regarding the survival risk associated with MPNST grade comes from small single-institution studies.^41,42^ Larger multi-institutional studies are needed to answer this question.

**Table 1.** Pathology definitions from the consensus meeting on pathology of NF1-associated atypical nerve sheath tumors, held in October, 2016, at the NCI/NIH, Bethesda, Maryland

Tumor	Definition
Plexiform neurofibroma	Neurofibroma replacing a nerve involving multiple nerve fascicles with EMA+ perineurial cells.
Neurofibroma with atypia	Neurofibroma with atypia alone (usually bizarre nuclei).
Atypical neurofibromatous neoplasm of uncertain biologic potential	Schwann Cell Neoplasm with 2/4 features: cytologic atypia, loss of neurofibroma architecture, hypercellularity, mitotic index >1/50 HPF but <3/10 HPF.
Low grade MPNST	Mitotic index 3–9/10 HPF and no necrosis.
High grade MPNST	Mitotic index of >10/HPF or 3–9/10 HPF with necrosis.

Given the lack of specific pathologic diagnostic criteria, the field would benefit from identification of more accurate biomarkers for this disease. Mutations in polycomb repressive complex 2 (*PRC2*)/PCR2 subunits (such as SUZ12) are observed in as many as 70% of MPNST, but not in benign PNs or AN.^14,15,43^ As a surrogate for *PR*C2/SUZ12 loss, loss of trimethylation at lysine 27 of histone 3 (H3K27me3), a known downstream target of SUZ12, can be quantified.^14,17,44–46^ Additionally, whole exome sequencing and subsequent immunohistochemistry studies identified β-III-spectrin in as many as 90% of MPNST.^20,47^ Future studies examining the utility of H3K27-me3 and β-III-spectrin immunohistochemistry are required to define their utility in the diagnosis of MPNST.

Furthermore, the decision to pursue a biopsy versus upfront resection of a suspected MPNST is another area of controversy. Sampling error within biopsy is a major point of contention, as it can lead to a false representation of the tumor. Within a single tumor, there may be areas of PN, ANNUBP, low grade MPNST, and high grade MPNST. As such, the diagnosis rendered by a biopsy may not be representative of the entire tumor, suggesting that upfront resection may be a more valuable diagnostic tool.

## Treatment of Localized Disease

Given that there are limited treatment options for MPNST and that most MPNST arise from a PN, there is a strong interest in prevention of malignant transformation. There are several case reports demonstrating efficacy of trametinib, an oral inhibitor of MEK 1/2,^48,49^ though the most promising data to date has been from the use of selumetinib, another oral selective inhibitor of MEK 1/2, in children who had NF1 and inoperable PN. In a phase I study, treatment with selumetinib resulted in confirmed partial response (≥30% decreases in tumor volume from baseline) in 17/24 children (71%).^50^ This response rate (RR) was confirmed in the recently presented phase II study.^51^ These children are still in active follow-up to determine if this treatment will prevent malignant transformation.

Once malignant transformation occurs, the mainstay of therapy for MPNST is local treatment. Complete surgical excision with negative margins remains the only proven curative treatment,^52,53^ though this is often not feasible due to tumor location or size. Biopsies are conducted in patients that might benefit from down-staging the tumor in order to make an unresectable tumor amenable to surgery, to obtain a diagnosis of malignancy prior to neoadjuvant chemotherapy or radiation therapy. However, there is limited published data available regarding the use of chemotherapy in the neoadjuvant setting for MPNST. The only prospective data comes from SARC006, a study in which unresectable or metastatic patients with sporadic or NF1-associated MPNST were treated up-front with chemotherapy. In this study, there were minimal responses with adriamycin and ifosfamide, with RR of approximately 17% (5/29 patients) in patients with NF1.^54^ A slightly higher RR (4/12 patients; 33%) was observed in patients with sporadic MPSNT. Our own institutional experience has been slightly different. Following neoadjuvant chemotherapy with epirubicin and ifosfamide, we observed decreased tumor size, including three partial responses (PR) and two patients with stable disease (SD), with a promising RR (3/5 patients; 60%) and clinical benefit rate (CBR = PR + SD; 100%).^55^ Furthermore, similar RR were observed for both NF1-associated and sporadic MPNST. Future well-designed and adequately powered prospective trials are needed to determine the true benefit of neoadjuvant chemotherapy for this subtype of sarcoma.

The use of adjuvant chemotherapy for MPNST has also been debated. Several studies have failed to show a survival benefit for chemotherapy in the treatment of MPNST.^56–58^ However, the most of these studies were small and retrospective, encompassing patients treated with different regimens, and often pooling data from multiple trials at multiple institutions. While most regimens include ifosfamide, the anthracycline used most often was doxorubicin. The only study that showed a survival benefit to adjuvant chemotherapy in soft tissue sarcomas, including MPNST, utilized epirubicin as the anthracycline. In this study, patients with high-grade sarcomas, including MPNST, were randomized to receive chemotherapy with five cycles of epirubicin and ifosfamide or standard follow-up. Randomization occurred following some form of local therapy (amputation, wide resection followed by radiation, or pre-operative radiation followed by surgery). The median overall survival was 75 months in the chemotherapy group compared to 46 months for the patients in the control arm (*P* = .03).^59^ A retrospective analysis at our own institution showed a similar overall survival advantage to this combination of chemotherapy in the adjuvant setting.^60^ Of note, one of the dose-limiting side effects of anthracyclines is cardiac toxicity. There are both preclinical and clinical data demonstrating that higher doses of epirubicin can be given with less risk of cardiac toxicity relative to doxorubicin. This reduced cardiotoxicity may partially explain why improved RR are seen with epirubicin.^61–63^ Future studies would be required to prospectively evaluate the utility of epirubicin-based chemotherapy in MPNST.

Adjuvant/neoadjuvant radiation therapy is another area of debate in the treatment of MPNST. While the role of radiation therapy is unclear for MPNST, it is often recommended for high-grade lesions or tumors greater than 5 cm.^64^ These recommendations are based on data for high grade soft tissue sarcomas as a group, in which radiation therapy has improved local control, but not overall survival.^65,66^ Neoadjuvant radiation may be useful for downsizing tumors to make surgery possible for otherwise unresectable lesions, and can lead to fewer long-term complications compared to adjuvant radiation.^67^ Of note, the risk of radiation-induced sarcomas may be greater in patients with NF1 relative to the general population. In one study, the incidence of second malignancies in patients with NF1-associated optic pathway gliomas (NF1-OPG) treated with radiation therapy was significantly higher compared to those who did not receive radiation. Almost 50% of patients who received radiation developed MPNST in the radiation field, while only 20% of patients who did not receive radiation developed MPNST. Interestingly, all of the NF1-OPG patients who developed MPNST in the radiation group received the treatment as children, suggesting that the greatest risk for the development of a secondary malignancy due to radiation occurs in childhood.^68,69^ In contrast, a retrospective analysis of the long-term outcomes of NF1-MPNST patients treated with radiation found that MPNST patients treated with radiation had no difference in outcome compared to patients with other soft tissue sarcomas.^70^ Future prospective studies are needed to better evaluate the potential risks of radiation in these tumors.

## Treatment of Metastatic Disease

Doxorubicin-based cytotoxic chemotherapy remains the standard of care treatment for unresectable or metastatic MPNST. RR for unresectable or metastatic disease range from 20% to 60%, depending on the study. When stable disease is taken into account, the CBR approaches 80%.^55–57^ While the highest RR are seen in regimens containing ifosfamide, there is no overall survival advantage to adding this drug, though there is increased toxicity.^71,72^ Despite initial responses, 5 years overall survival rates remain low, and initial responses to therapy are usually short-lived, followed by rapid progression and death. As such, fewer than 40% of patients with unresectable or metastatic disease will live beyond 1 year post-diagnosis.

Given the lack of effective therapies, clinical trials for MPNST are encouraged. To date, there have been several clinical trials using targeted agents in an attempt to find more promising therapies. Table 2 shows an updated summary of clinical trials to date.^73–75^ The first targeted agent used in a trial designed specifically for MPNST involved the epidermal growth factor receptor (EGFR) inhibitor, erlotinib. This phase II trial was based on preclinical work in which *Nf1;p53*-deficient murine MPNST cell lines showed overexpression of EGFR, response to EGF stimulation, and decreased growth following EGFR inhibition.^76^ Despite these promising preclinical results, no activity was demonstrated in the clinical trial.^77^ There have been several other trials based on similar preclinical outcomes, including sorafenib,^78^ imatinib,^79^ dasatinib,^80^ and alisertib.^81^ Unfortunately, none of these drugs demonstrated any activity in human clinical trials. Two recent studies, also based on preclinical data using the *Nf1;p53*-deficient mouse model, were performed in patients with NF1-associated and sporadic MPNST using a different approach. SARC016 evaluated the combination of bevacizumab (angiogenesis inhibitor) and RAD001 (mTOR inhibitor),^82,83^ while SARC023 evaluated the combination of ganetespib (Hsp90 inhibitor) and sirolimus (mTOR inhibitor) in patients with both sporadic and NF1-associated MPNST.^84^ These studies revealed a poor overall survival for patients with NF1-MPNST (~5 months), strongly underscoring the need for better therapies. A recently reported phase I study evaluating the combination of pexidartinib (tyrosine kinase inhibitor) and sirolimus in MPNST and other soft tissue sarcomas demonstrated sustained SD in 5/6 patients and an acceptable safety profile, triggering expansion into a phase II trial for patients with MPNST.^85^ Currently, SARC031 is evaluating combined MEK/mTOR inhibition (NCT03433183), further emphasizing the need for trials that combine inhibitors with preclinical justification in MPNST. Finally, immunotherapy is also starting to be explored in the treatment of MPNST. There are case reports showing response in metastatic disease^86^; however, prospective trials are needed to adequately explore the role of immunotherapy in the treatment of MPNST.

**Table 2.** Clinical trials of targeted therapies for MPNST

Therapy	Molecular targets	No. of MPNST	Study design and population	Response	References
Erlotinib	EGFR	20	Phase II study in MPNST	No objective responses, 1 stable disease	72
Sorafenib	VEGFR, RAF, PDGFR	12	Phase II study in soft tissue sarcomas	No objective responses	73
Imatinib	c-KIT, PDGFR, VEGFR	7	Phase II study in 10 subtypes of sarcoma	No objective responses, 1 stable disease	74
Dasatinib	c-KIT, c-SRC	14	Phase II study in bone and soft tissue sarcomas	No objective responses	75
Alisertib	Aurora Kinase A	10	Phase II study in advanced sarcomas	No objective responses	76
Bevacizumab/RAD001	VEGF/mTOR	25	Phase II study in MPNST	2 stable disease, 1 partial response after 2 cycles that progressed after cycle 4	77, 78
Ganetespib/Sirolimus	HSP90/mTOR	20	Phase I/II study in MPNST	Not fully reported	79
Pexidartinib/Sirolimus	c-KIT, PDGFR, CSF1R/mTOR	6	Phase I study in MPNST, PVNS, and other sarcomas	5 stable disease	80
Selumetinib/Sirolimus	MEK/mTOR	21	Phase II study in MPNST	Enrolling	N/A

c-KIT, stem cell factor receptor; CSF1R, colony stimulating factor 1 receptor; c-SRC, cellular SRC kinase; EGFR, epidermal growth factor receptor; HSP90, heat shock protein 90; mTOR, mammalian target of rapamycin; PDGFR, platelet derived growth factor receptor; PVNS, pigmented villonodular synovitis; RAF, rapidly accelerated fibrosarcoma; VEGF, vascular endothelial growth factor (ligand); VEGFR, vascular endothelial growth factor receptor.

## Future Directions

The vast majority of trials have focused on RAS effectors, such as the PI3K/AKT/mTOR or RAS/REF/MEK pathways, and unfortunately have failed to show clinical benefit, illustrating the need to identify other pathways as important targets for MPNST drug development. Within the last year, several novel targets have been identified.

Gene-based therapies that focus on alterations of the polycomb repressive complex 2 (PRC2) are one area of interest. Mutations in components of the PRC2 complex, including embryonic ectoderm development (*EED*), suppressor of zeste 12 protein homolog (*SUZ12*), and retinoblastoma-binding protein 4/7 (RBBP4/7) result in an epigenetic loss of H3K27me3, which can be evaluated by immunohistochemistry. As previously mentioned, these mutations are observed in as many as 70% of MPNST, and other components of the PRC2 complex (such as EZH1 and EXH2) have been shown to be elevated in MPNST, making them an appealing target for drug development.^14,15,43^ PRC2 inactivation results in loss of H3K27me3, and a subsequent increase in acetyl groups,^87^resulting in recruitment of BET proteins, such as BRD4, to the chromatin. This results in MPNST sensitivity to BRD4 inhibitor JQ1.^14,88,89^ Additionally, utilizing EZH2 inhibitors, such as tazemetostat could be considered in combination therapies targeting the PRC2 complex.

Tyrosine Kinase 2 (*TYK2*) has recently been identified as being mutated, and the subsequent protein highly expressed, in the majority of MPNST.^90^ TYK2 is a part of the Janus Kinase (JAK) pathway. JAKs activate the signal transducers and activator of transcription (STAT) family of transcription factors, which induce transcription of cell proliferation and cell death signaling proteins, such as cyclin D, Bcl-2, and Bcl-x.^91,92^ Several groups have illustrated the importance of the STAT pathway in MPNST.^87,93,94^ Furthermore, our recent study has identified the importance of TYK2 in the progression of MPNST, and supports our previous work identifying this aberration within clinical samples.^90,95^ We demonstrated that TYK2 knock-down leads to decreased phosphorylation of the STAT proteins, resulting in decreased levels of Bcl-2 and promotion of cell death both in vitro and in xenografts in vivo. This finding allows for the exploration of new therapeutic avenues within the JAK/STAT pathway, either alone or in combination with other compounds.

Finally, the HIPPO pathway has been identified as an important possible target in MPNST. Recently, hyperactivity in the HIPPO pathway was found to result in constitutively active YAP/TAZ oncoproteins in both sporadic and NF1-associated MPNST.^96^ This leads to stem cell-like characteristics, including resistance to chemotherapy.^97^ Targeting the HIPPO/YAP/TAZ pathway in combination with PDGFR/RAF1 signaling decreased tumor growth in mouse models and human MPNST cell lines. Clinically, PDGFR/RAF1 inhibitors such as imatinib and sorafenib have failed to elicit any type of efficacy in patients as single agents.^78,79^ However, if utilized in combination with verteporfin,^98^ an FDA-approved drug that has been shown to inhibit YAP via upregulation of the tumor suppressor protein 14-3-3σ, may lead to an improved response.

Our knowledge of the genetic profile and tumor evolution of MPNST has greatly increased over the past decade. Additionally, new pathways that have a role in the development and progression of these tumors have been identified. Further understanding of the genomics, epigenetics, signaling pathways, and metabolic alterations in MPNST, coupled with better comprehension of the crosstalk between tumor and surrounding microenvironment, will lead to more novel combinations of targeted therapies that have increased efficacy and specificity in these tumors.
